# Generation of a galactic chronology with impact ages and spiral arm tangents

**DOI:** 10.1038/s41598-024-56397-4

**Published:** 2024-03-09

**Authors:** Michael Gillman, Rui Zhang

**Affiliations:** 1 School of Environment, Earth and Ecosystem Sciences, Open University, Walton Hall, Milton Keynes, MK7 6AA UK; 2 Institute of Energy, Peking University, Beijing, 100871 China; 3 School of Earth and Space Sciences, Peking University, Beijing, 100871 China

**Keywords:** Impact periodicity, Spiral arm patterns, Mass extinctions, Geological cyclicity, Stratigraphy, Palaeoclimate

## Abstract

Resolving the role of galactic processes in Solar System/Earth events necessitates a robust temporal model. However, astrophysical theory diverges with models varying from long-lasting spiral density waves with uniform pattern speeds and arm structures to others with fleeting and unpredictable features. Here, we address those issues with (1) an analysis of patterns of impact periodicity over periods of 10 to 250 million years (Myr) using circular statistics and (2), an independent logarithmic spiral arm model fitted to arm tangents of 870 micron dust. Comparison of the impact periodicity results with the best-fit spiral arm model suggests a galactic period of 660 Myr, i.e. 165 Myr to pass from one arm to the next in a four spiral arm model, with the most recent arm passage around 52 million years ago (Ma). The oldest impact ages imply that the emerging galactic chronology model is robust for at least the last 2 Gyr. The arm-passing time is consistent with spectral analyses of zircons across 3 Gyrs. Overall, the model provides a temporal framework against which to test hypotheses of galactic mechanisms for global events such as mass extinctions and superchrons.

## Introduction

Galactic processes have been strongly implicated as drivers of multi-million period cycles of species turnover and therefore potential contributors to extinction events^1–4^. Raup and Sepkoski^5^ considered passage of the Solar System through the spiral arms of the Milky Way as a possible driver of the 26 Myr cycles detected in marine fossil data. This was in turn linked to increased comet flux through the galactic arms^6^ and the Alvarez hypothesis positing that an impact triggered the end-Cretaceous extinction event and potentially other extinctions^7^. Shoemaker suggested passage through the spiral arms occurred with a frequency in the order of 100 million years*.* Large terrestrial impacts cluster in discrete episodes but the periodicity of these clusters is unclear^8–10^.

The Sepkoski (and similar) fossil datasets have been revisited and yielded statistically significant periodicity, notably, a 62 ± 3 Myr period^11^ and 27 Myr periodicity within the 62 Myr signal^12^ – see also their discussion of criticisms of periodicity assessment. 27.3, 32 and 36.9 Myr cycles have also been detected in non-marine fossil data^4^, with a 27.5 Myr pulse across multiple geological events over the past 260 Myr^13^. Rohde and Muller^11^ considered seven possible geophysical drivers, including passage of the Solar System through the spiral arms (involving perturbations from molecular clouds) and oscillations around the galactic plane, noting the possibility of reduced oscillation half-periods closer to the arm centre. Long-term periodicity, exceeding 10 Myr cycles, has also been detected in a range of geophysical phenomena including magnetism/superchrons^14,15^ using superchron data^16^, ice ages^17,18^, δ^18^O flux as a proxy for temperature^19^, large igneous provinces^20^, various isotope signatures associated with mantle activity^21^, zircon grains and isotopes indicating mantle activity^22,23^, strontium isotopes^24^, sedimentation rates^24–26^ and sea level^27^.

Resolution of the role of galactic processes in Solar System/Earth events requires a robust temporal model. Such models of the passage of the Solar System through the spiral arms have been developed using various data, methodologies, and assumptions^15,17,28–32^. An important assumption is that the arm structure and arm passage time is consistent over sufficiently long time periods to encompass geological and Solar System events (up to a maximum of 4.57 Ga). There is a divergence in astrophysical theory on this assumption, with models differing from long-lived spiral density waves with consistent pattern speeds and arm structures to those with transient and unpredictable properties^33^.

A key variable for unlocking the effects of spiral arms on events on Earth is the net time for the Solar System to pass through the four spiral arms of the Milky Way and return to the same point with respect to the spiral arms (defined as the galactic period^15,32^), noting that both the Solar System and arms are rotating in the same direction at different speeds. More recently, the time for the Solar System to pass from one spiral arm to the next has been estimated as 157.5 ± 10 Myr based on astrophysical measurements^34^. The model is based on four equally separated arms and so the galactic period is estimated as 157.5 × 4 = 630 ± 40 Myr. This method has provided a more precise estimate than comparisons of Solar System speed with arm pattern speed due to the high variation in assessments of the latter (e.g. 18.1–30 km s^–1^ kpc^–1^ in the review by Ref.^35^, which overlaps with their own estimate). The Earth’s evolution has been intricately linked to the wandering of the Solar System through these spiral arms, whereby phenomena such as deposition of energy resources, emergence of life, ingress of cosmic dust, stellar detonations, and production of heavy elements take place in a cyclic pattern spanning ca. 150 Myr^10^.

Here we determine the periodicity of impacts of different sizes and compare their mean position with the position of spiral arms from an independent model. This leads to an estimate of the galactic period assuming a four spiral arm model of the Milky Way. The resulting predictions of passage time between arms and location of arms then facilitates comparison with the timing of global events, illustrated here with superchrons, black shale-large igneous province co-occurrences and mass extinctions.

## Data and methods

### Impact data

Impacts with crater diameters of ≥ 20 km and age error of ≤ 5 Myr (impact data from Refs.^36,37^ and their supplementary data) were divided into three (nested) size categories for analysis (> 20 km n = 25, > 39 km n = 14 and > 70 km diameter, n = 8, Table 1).

**Table 1:** Impacts with crater diameter greater than 20 km and errors of 5 Myr and less.

Impact	Crater diameter (km)	Age (Ma)	Error (Myr)	Modulo age (Ma, 165 Myr divisor)
Vredefort	300	2023	4	43
Sudbury	200	1849.53	0.21	34.53
Chicxulub	180	66.038	0.098	66.038
Manicouagan	100	215.4	0.16	50.4
Popigai	100	36.63	0.92	36.63
Chesapeake Bay	90	34.86	0.32	34.86
Puchezh-Katunki	80	195.9	1.1	30.9
Siljan	75	380.9	4.6	50.9
Morokweng	70	146.06	0.16	146.06
Yarrabubba	70	2229	5	84
Kara	65	75.34	0.66	75.34
Montagnais	45	51.1	1.6	51.1
Araguainha	40	254.3	3.5	89.3
Lake Saint Martin	40	227.8	0.9	62.8
Carswell	39	481.5	0.8	151.5
West Clearwater	36	286.2	2.6	121.2
Manson	35	75.9	0.1	75.9
Rochechouart	32	206.92	0.32	41.92
Hiawatha	31	57.99	0.54	57.99
Mistastin	28	37.83	0.05	37.83
Kamensk	25	50.37	0.4	50.37
Boltysh	24	65.39	0.16	65.39
Ries	24	14.808	0.038	14.808
Haughton	23	31.04	0.37	31.04
Lappajärvi	23	77.85	0.78	77.85

Details from Ref.^37^. Restricted to impacts with isotope age determination (i.e. not stratigraphic location). The Araguainha impact with 40 km crater does not have a single agreed age^37^ but it has an average age of 254.3 ± 3.5 Ma across several studies^36^.

### Circular statistics

Temporal clustering of impacts was explored with changing periods from 10 to 250 Myr using the modulo age of impacts (modulo age is the remainder after dividing by the divisor). Impacts that cluster at certain periods (given by the modulo divisor) will have similar modulo ages. For example, impacts with ages of 56, 157 and 255 Ma will have modulo ages of 56, 57 and 55 Ma with a modulo divisor of 100 Myr. With a consistent relative speed of Solar System to arm pattern, the interpretation of the clustering of modulo ages is that the impacts are at similar spatial positions, e.g. with respect to the arm center. The arm-passing time would be given by the modulo divisor, and the galactic period by four times the arm-passing time. (Impacts may also cluster in absolute age, e.g. Kamensk and Montagnais which are within error, Table 1). Modulo age cannot be used directly for assessing clustering because values close to 0 and close to the maximum modulo age (for a given divisor) will have average values close to the midpoint of the modulo age. For example, 3, 99, 195 and 203 will have modulo ages of 3, 99, 95 and 3 with a divisor of 100 and an average of 50. This issue is solved by the use of circular statistics which are designed to overcome the ‘wrap-around’ problem, e.g. angles of 359 and 1 degree are very close (separated by two degrees and not 358 degrees).

The modulo ages were converted to fractions of the modulo divisor and then to radians. The age equivalent in radians could then be analyzed with the circular statistics package “CircStats” in R^38^. The mean direction and average radius (rho) were determined, along with the Kuiper statistic value, which measures the fit to a uniform distribution. The average radius values vary from 0 (completely uniform, radius distances sum to zero) to 1 (identical radius values for all impacts, i.e. complete clustering of values for a given divisor, e.g. 46, 146 and 246 with a modulo divisor of 100). High radius values are associated with high Kuiper statistic values (critical values of 1.747 for P < 0.05 and 2.001 for P < 0.01). N = 8 is the minimum recommended sample size for the Kuiper test.

### Modeling arm location

Independent from the impact data, a model of location of spiral arms was constructed. The model assumed four equally separated arms, described by a logarithmic spiral. The log-spiral model follows Eq. (3) in Ref.^39^ with the start azimuth angle being referred to here as rotation angle. The model was fitted to six arm section tangents across four arms using the 870 micron dust data^40^ and checked against^41^, varying the pitch and rotation angles to get the best overall fit (minimum average distance from the model arms to the tangents). Once the best fit of the four logarithmic spirals was obtained, the timing of interception of arms (assuming a constant net velocity of the Solar System relative to the arms) could be determined from the galactic period given a Solar galactocentric radius of 8.178 kpc^42^. This result is based on the hypothesis that frequency of large impacts will increase during arm passage.

### Comparison with events on Earth

The significant periodicity results from the impact analyses provide the opportunity to make comparisons with and between events on Earth using the modulo ages. Three groups of events were chosen to illustrate a variety of processes across the last 1.6 Gyr. These comprised the eight most severe extinctions^43^ (Table S1), the last five superchrons (Table S2) and the five ages during which there was believed to have been a robust or likely temporal connection between black shales and large igneous provinces during the ‘boring billion’^44^.

## Results

### Impact analysis

**Figure 1:**
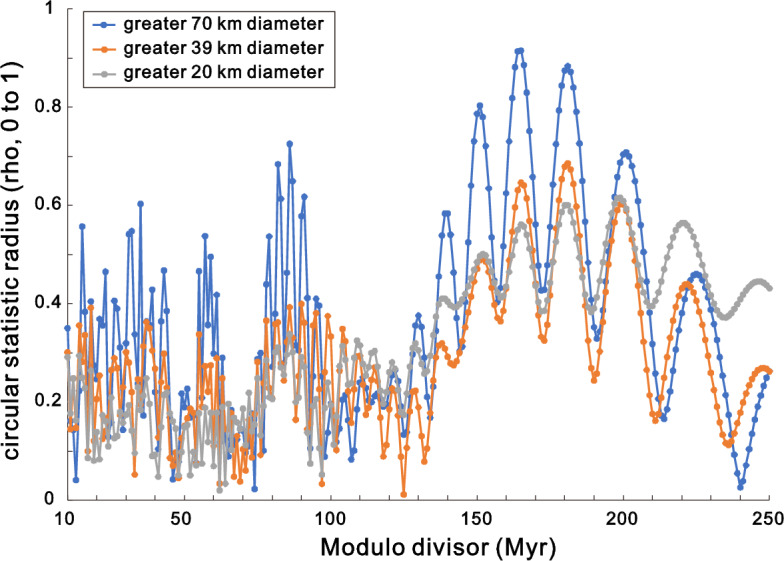
Change in circular statistic radius with modulo divisor (period, Myr).

**Figure 2:**
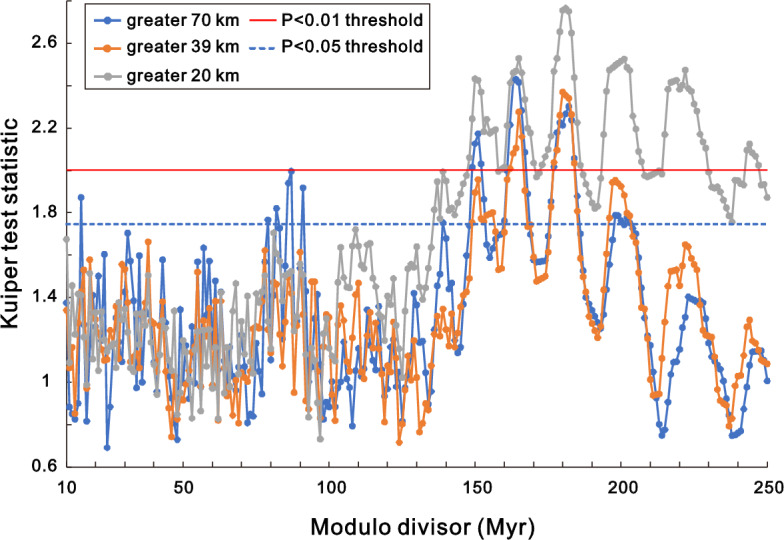
Change in Kuiper test statistic with modulo divisor (period, Myr). Note: The significance around 80 Myr occurs only for the largest impacts with a small sample size (n = 8). Three of the impacts have ages less than the modulo divisor (34.86, 36.63 and 66.038 Ma), i.e. their modulo ages are the same as their absolute ages. The average of their absolute ages (45.8 Ma) is equal to the average modulo ages of the other five largest impacts with a modulo divisor of either 164.28 Myr or 164.28 divided by 2 = 82.14 Myr.

The largest impacts (> 70 km crater diameter) had significant P < 0.01 modulo divisor peaks of 151, 165 and 181 Myr (peak values were taken from the radius values, Fig. 1 – mostly identical or very close to the Kuiper statistic peaks, Fig. 2). The highest radius value for the largest impacts occurred at 165 Myr. Impacts greater than 39 km had P < 0.01 peaks at 165 and 181 Myr, with 181 Myr having the highest value. The third group of impacts, covering all those analyzed here (Table 1), had a maximum peak at 199 Myr, with five peaks at P < 0.01.

The mean direction from the circular statistics was contrasted with the predicted arm position for each size category across the four peaks from 151 to 201 Myr (Table 2). The average impact age, calculated from the mean circular statistic position, was closest to the arm position with a period of 165 Myr and therefore a galactic period of 660 Myr (contrast the average age of 52.6 and 51.6 Ma for > 39 km and > 20 km diameter with 52.1 Ma for the arm position at 660 Myr galactic period, Table 2).

**Table 2:** Summary statistics for four peaks in circular statistic radius across three impact size categories.

	Impact category	Peak	Modulo divisor (period)	Galactic period	Radius	Kuiper test statistic	P value	Mean age (Ma)
	> 70 km	1	151		0.803	2.173	< 0.01	52.61
	> 39 km	1	152		0.494	1.774	< 0.05	58.74
	> 20 km	1	152		0.501	2.370	< 0.01	53.90
Arm positions		1	151	604				47.63
		1	152	608				47.95
	> 70 km	2	165		0.915	2.415	< 0.01	43.13
	> 39 km	2	165		0.646	2.276	< 0.01	52.63
	> 20 km	2	165		0.561	2.529	< 0.01	51.59
Arm position		2	165	660				52.05
	> 70 km	3	181		0.884	2.267	< 0.01	34.15
	> 39 km	3	181		0.686	2.355	< 0.01	43.26
	> 20 km	3	181		0.600	2.766	< 0.01	48.23
Arm position		3	181	724				57.10
	> 70 km	4	201		0.708	1.744	> 0.05	22.70
	> 39 km	4	199		0.603	1.939	< 0.05	37.45
	> 20 km	4	199		0.616	2.506	< 0.01	45.80
Arm positions		4	199	796				62.78
		4	201	804				63.41

**Figure 3:**
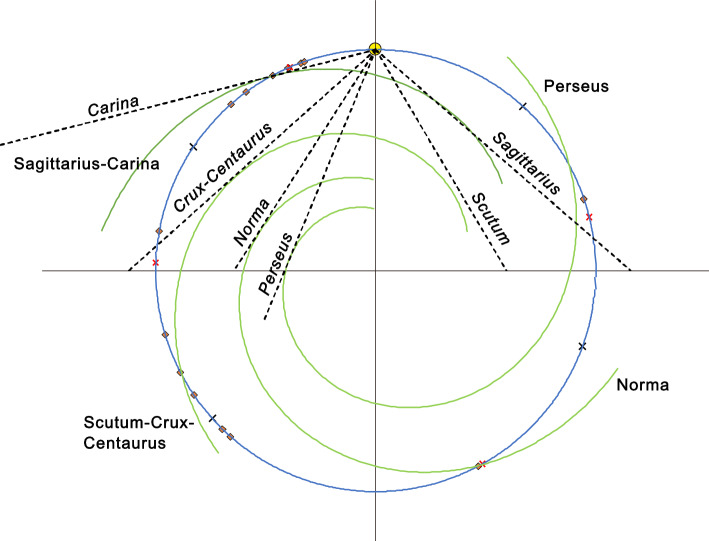
Distribution of spiral arms and largest impacts. The galactic orbit is depicted as a blue circle with the present position of the Solar System at the top (yellow filled circle). Both the Solar System and the arms move clockwise in this view. Impacts (diamonds) are those with crater diameters of 40 km and greater (Table 1). Symbol x indicates start (black) and end (red) of arms in Reid et al.^45^ (Fig. S1). Spiral arms (labelled) are modelled as logarithmic spirals with equal separation. The straight dashed lines are 870 micron dust tangents (italicized labels).

The mean age (Ma) is calculated from the mean direction for each peak and size category. This is compared with the age of the Sagittarius-Carina arm (arm position) using the galactic period (4 × modulo divisor) and position in Fig. 3.

### Arm location

The best fit was 13.8 degrees pitch and 41.57 degrees rotation (analyzed to four significant figures for pitch and rotation). The only apparent deviation, with the spiral about 0.36 kpc inside the tangent, was the Scutum (right-hand) section of the Scutum-Crux-Centaurus arm (Fig. 3). This may reflect a kink in this arm^45^. The start and end locations of the spiral arms^45^ (Fig. S1), along with impacts > 39 km, are also shown (Fig. 3).

### Comparison with events on Earth

**Figure 4:**
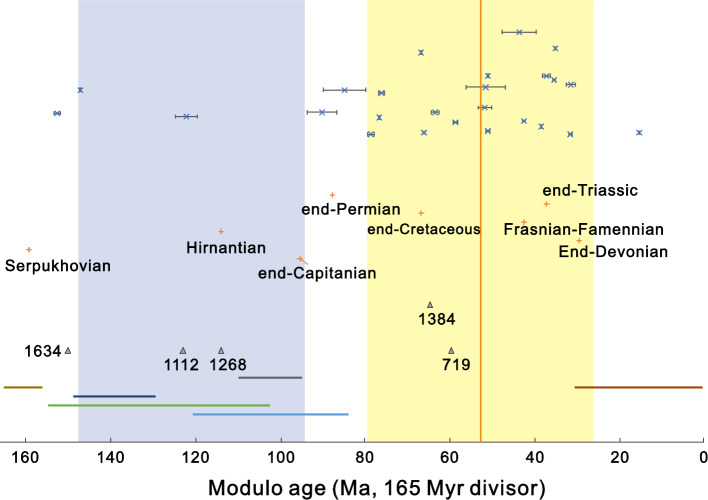
Impact modulo ages contrasted with mass extinctions, black-shale and LIP co-occurrences and superchrons. The top part of the figure gives impacts (x, ± 1 SE) with the vertical position scaled by log_10_ crater diameter (largest at the top of the figure). The mass extinctions ( +) are also scaled from most to least severe (most severe at top) and labelled (Table S1). The black-shale and LIP co-occurrences are those listed in Ref.^44^ as robust (1384 label) or likely (other four). The numerical labels are representative absolute ages (Ma). The bottom part of the figure shows the duration of the five most recent superchrons (Table S2) with oldest at top. The Maya superchron is split across two regions of the modulo age scale. The left-hand rectangle encompasses all events within the locations where two or more superchrons overlap. The right-hand rectangle covers the same duration but is located around the arm centre indicated by the 870 micron dust (52 Ma, vertical line).

The modulo ages of impacts, mass extinctions, superchrons and the large igneous province (LIP) and black shale co-occurrences were contrasted using a modulo divisor of 165 Myr (Fig. 4). The impacts and global events are shown in relation to the calculated 870 micron dust age. Overlapping superchrons occur away from the arm centre, with two or more superchrons occurring from 147.7 to 94 Ma (modulo ages, rectangle on left of Fig. 4). Within this region there are two impacts. A similar duration for either side of the arm centre encompasses 19 impacts (a significantly higher proportion, P < 0.001, binomial test in R^46^, consistent with the significant departure from uniform distribution detected with the Kuiper test).

The largest extinction at the end-Permian sits between the superchron and arm regions, within error of the modulo age of the oldest impact (Yarrabubba) and absolute age of Araguainha. The modulo age of Yarrabubba with a divisor of 4 × 165 Myr, i.e. the galactic period, is 249 Ma indicating that Yarrabubba and the end-Permian are predicted to have occurred in the same region of the same arm (Scutum). The end-Cretaceous, estimated to be 14 Myr prior to the 870 dust tangent, is close to the modulo age of the Xiamaling black shale formations (peak age 1384 Ma^44^, equivalent to 64 Ma) and Mutare and Franklin LIPs (724 Ma equivalent to 64 Ma^44^ and 719 Ma equivalent to 59 Ma^47^). Both of these modulo ages are also equivalent to the modulo age with the galactic period as divisor, i.e. they are predicted to occur in the same arm (Sagittarius-Carina) as the end-Cretaceous. The end-Triassic, with the second highest overall rank (Table S1), overlapping the largest LIP (CAMP), has a modulo equivalent of 36.6 Ma, placing it within error of the Popigai impact (Table 1).

## Discussion

The significant period (P < 0.01) values ranged from 149 to 184 Myr for the > 70 km and > 39 km impact crater categories (Fig. 2). The only shorter periods occurred at half the longer periods for the largest category (79 to 91 Myr, P < 0.05) and one spike at 15 Myr (P < 0.05). All the > 39 km below 149 Myr period are not significant (P > 0.05). The lack of signal for periods less than 50 Myr agrees with Ref.^9^ analysis of 26 impacts (some of which are included here and some of which have updated ages). Note the similarity of the radius in their Fig. 3 (R-statistic) with the radius values here (Fig. 1). Increase and then decrease to similar radius values for > 70 km and > 39 km across the 10 to 250 Myr range of periods suggests that this is not an artefact of sampling. In contrast, higher radius and Kuiper values for > 20 km category at larger periods (Figs. 1 and 2) may partly reflect an artefact of higher fraction of low absolute ages for smaller impacts.

Comparing the mean direction of impact peaks with arm analyses (Table 2) reveals 165 Myr to be closest and is within error of an estimate based on astrophysical processes^34^. An important astrophysical implication of the impact model is the spiral arm pattern speed. A galactic period of 660 Myr with Solar System speed of 233.4 ± 1.5 km/s^48^ and galactic radius of 8.178 kpc^42^ corresponds to a pattern speed of 19.23 km s^–1^ kpc^–1^, towards the lower end of the range^35^. Inclusion of Vredefort and Sudbury with absolute ages of 2023 Ma and 1849.53 Ma (Table 1) and modulo ages of 43 and 34.53 Ma with a 165 Myr period suggest that these patterns may be consistent over the past 2 Gyr. Furthermore, the modulo ages with a galactic period divisor of 660 Myr gives ages of 43 and 529.53 for Vredefort and Sudbury, placing them in the Sagittarius-Carina and Perseus arms respectively. Yarrabubba, the oldest impact, would be placed within the Scutum-Crux-Centaurus arm with a modulo age of 249 Ma (using a 660 Myr divisor and 84 Ma with 165 Myr divisor).

The estimate of 165 Myr for the arm-passing time differs from a previous estimate of 188 Myr using superchrons^15^. Superchrons provide a potential terrestrial marker of inter-arm passage^49,50^ but there is no agreed galactic causal mechanism. Furthermore, the identification of the older superchrons is dependent on the sampling window^16^. Considering the five most recent superchrons for which there are reasonable stratigraphic data (Table S2), and assuming that the arm locations at 52.05 + n 165 Ma (Table 1, 165 Myr peak), then we find there is no overlap with the superchrons (Fig. 4). Thus, the distribution of superchrons over the last 1.1 Gyr is consistent with positions away from the arm locations.

The predicted ages of 52, 217, 382 and 547 Ma for the four most recent arm passages can be contrasted with ages predicted from the galactic period of 660 Myr and the distribution of arms in galactic maps^45^ (Fig. S1). The midpoints of the arm ages are 72 Ma (range 42–103 Ma), 202 Ma (161–242 Ma), 421 Ma (383–458 Ma) and 552 Ma (521–583 Ma). The Norma predicted age from the study here is at the lower end of the arm range^45^. Otherwise, the three estimates are consistent (Fig. 3). The zircon data^23^ cover multiple galactic periods and are therefore expected to account for any variation within the galactic period. Periodicities in zircon production assessed across 3 Gyr, and detected to 95% confidence with at least three out of four methods, were 17, 20, 31, 44, 57, 69, 100, 160, and 220 Myr^23^. The 160 Myr period agrees with the average arm-passing time. Focusing on the other largest periods, there is a possibility that some are simple integer fractions of the galactic period, i.e. potential harmonics, e.g. 220 × 3 = 660 Myr, 44 × 15 = 660 Myr and 31 × 21 = 651 Myr. The 660 Myr period is similar to the supercontinent cycle of approximately 600 Myr^22,26^. 168 Myr and 198 Myr Hafnium isotope periodicities have been linked to galactic arm crossing^51^ and agree with the range of peaks identified here.

Ascribing galactic causes to particular terrestrial events needs to be undertaken systematically (ideally statistically) across the full range of events (as with all of the largest impacts considered here) and with consideration of the variety of possible mechanisms. This may be on the back of previous analyses and correlations in Earth system processes^26^. For example, the exploration of potential links between black shales and igneous provinces during the ‘boring billion’ identified one robust link (around 1380 Ma) and four likely links (around 720, 1100, 1270 and 1650–1620 Ma^44^). Within the 1380 group, the Black shale Xiamling formation with a weighted mean of 1383 ± 2 Ma would be equivalent to 1383–660 = 723 Ma and 723–660 = 63 Ma. Therefore, this links to one of the four likely black shale-LIP combinations around 720 Ma (including the Mutare and Franklin LIPs) and then to Chicxulub/Deccan and the end-Cretaceous. One galactic cycle on from 1383 Ma gives 1383 + 660 = 2043 Ma, i.e. the same arm passage that includes the Vredefort impact. Sagittarius-Carina is therefore seen to have potential impact/black shale/LIP markers across its last four arm passages.

## Conclusion

The consistency of the 165 Myr impact signal with spiral arm positions, and support from recent astrophysical estimates and zircon analyses, suggests spiral arm passage to be a strong contributor to impact periodicity. If so, this should form the basis of a chronology from which to interrogate the galactic contribution to terrestrial phenomena. The suggestion is that arm passage not only increases the frequency of large impacts, but also creates conditions that increase the probability of large igneous provinces and black shale formation, thereby directly and indirectly increasing the likelihood of mass extinctions. Interpretation of the periodicity results and investigation of causal mechanisms are dependent on development of astrophysical and Earth science knowledge. However, the growing coincidences of temporal signals across different phenomena are indicating that seeking galactic solutions to Earth system processes may be a fruitful line of enquiry.

### Supplementary Information

Supplementary Information.

## Data Availability

All data analyzed during this study are included in this published article and its supplementary information file.
