# Supplementary material for: Generation of a galactic chronology with impact ages and spiral arm tangents
Source: Sci Rep. 2024 Mar 9;14:5790. doi: 10.1038/s41598-024-56397-4 (PMC10924879; doi:10.1038/s41598-024-56397-4)
Supplement: Supplementary file 1 — Supplementary Information. [file 41598_2024_56397_MOESM1_ESM.pdf]

Generation of a galactic chronology with impact ages and spiral arm tangents

Michael Gillman [a], Rui Zhang [b, c, *]

[a] School of Environment, Earth and Ecosystem Sciences, Open University, Walton Hall, Milton Keynes, MK7 6AA, UK

[b] Institute of Energy, Peking University, Beijing 100871, China

[c] School of Earth and Space Sciences, Peking University, Beijing 100871, China

* Corresponding author: Rui Zhang. Email: ruizhangxu@pku.edu.cn

Supplemental Material

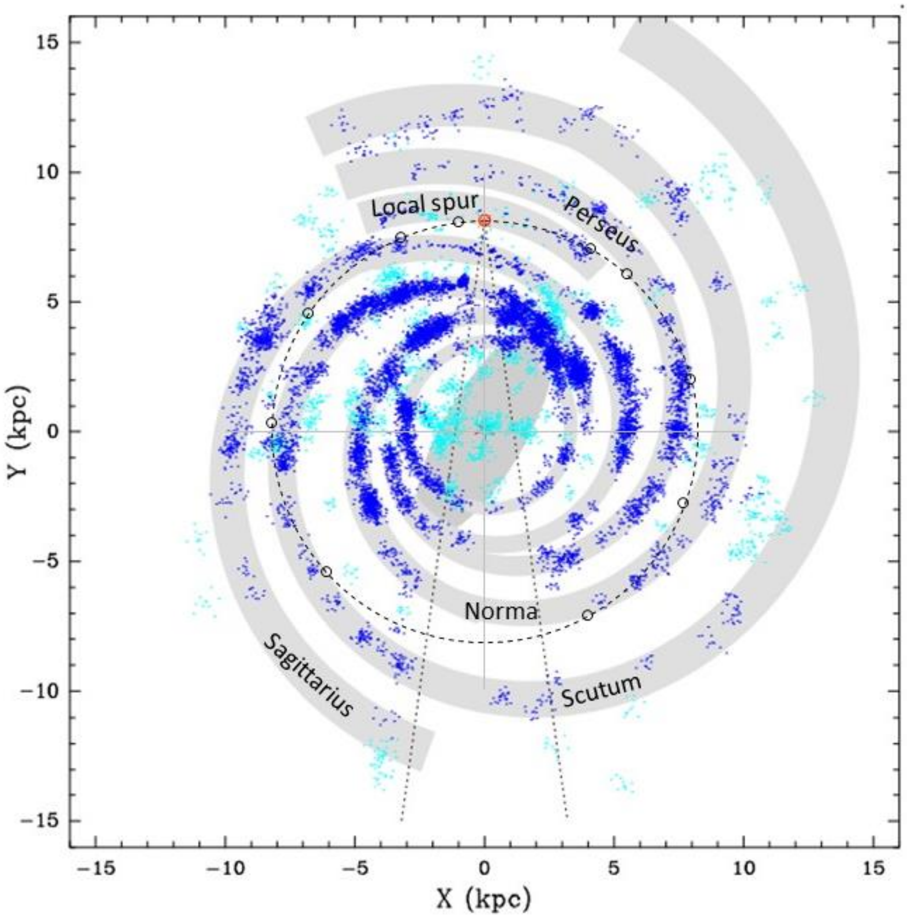

Figure S1. Locations of arms and local spur on galactic orbit of our Solar System. Overlaid on Figure 10 of Reid et al (2019). Symbols overlaid to approximately half edge. Entry angles (clockwise) 318° Perseus, 250° Norma, 132° Scutum, 56° Sagittarius and 7° local spur. Exit angles 284° Perseus, 209° Norma, 88° Scutum, 23° Sagittarius and 330° local spur.

Table S1. Mass extinction ages and references. First column is average rank of ecological and taxonomic-severity in Table 9 of McGhee et al (2013). The top eight extinctions are included here.

Average rank	Extinction event	Age (Ma)	Error	Comment	References
1	End-Permian	251.902	0.024	terrestrial extinction may be 252.3 Ma, Fielding et al 2019	Burgess et al (2014)
2.5	End-Triassic	201.564	0.015		Blackburn et al (2013)
3.5	End-Cretaceous	66.016	0.05		Keller et al (2020)
4.5	Late Devonian (Frasnian)	371.87	0.092	Frasnian-Famennian boundary	Da Silva et al (2020)
5	Hirnantian	442.99	0.17	analysed age within Hirnantian	Ling et al (2019)
5.5	End Devonian (Famennian)	358.93	0.2	midpoint of weighted means above and below Hangenberg shale, error is largest of two values	Myrow et al (2014)
6	Serpukhovian	323.2	0.4	may be earlier in Serpukhovian	Stratigraphic boundary, Cohen et al (2013)
7	Capitanian	259.51	0.21	may be earlier in Capitanian	Stratigraphic boundary, Cohen et al (2013)

References

Blackburn, T.J., Olsen, P.E., Bowring, S.A., McLean, N.M., Kent, D.V., Puffer, J., McHone, G., Rasbury, E.T. and Et-Touhami, M., 2013. Zircon U-Pb geochronology links the end-Triassic extinction with the Central Atlantic Magmatic Province. *Science*, *340*(6135), pp.941-945.

Burgess, S.D., Bowring, S. and Shen, S.Z., 2014. High-precision timeline for Earth's most severe extinction. *Proceedings of the National Academy of Sciences*, *111*(9), pp.3316-3321.

Da Silva, A.C., Sinnesael, M., Claeys, P., Davies, J.H., de Winter, N.J., Percival, L.M.E., Schaltegger, U. and De Vleeschouwer, D., 2020. Anchoring the Late Devonian mass extinction in absolute time by integrating climatic controls and radio-isotopic dating. *Scientific reports*, *10*(1), p.12940.

Fielding, C.R., Frank, T.D., McLoughlin, S., Vajda, V., Mays, C., Tevyaw, A.P., Winguth, A., Winguth, C., Nicoll, R.S., Bocking, M. and Crowley, J.L., 2019. Age and pattern of the southern high-latitude continental end-Permian extinction constrained by multiproxy analysis. *Nature communications*, *10*(1), p.385.

Keller, G., Mateo, P., Monkenbusch, J., Thibault, N., Punekar, J., Spangenberg, J.E., Abramovich, S., Ashckenazi-Polivoda, S., Schoene, B., Eddy, M.P. and Samperton, K.M., 2020. Mercury linked to Deccan Traps volcanism, climate change and the end-Cretaceous mass extinction. *Global and Planetary Change*, *194*, p.103312.

Ling, M.X., Zhan, R.B., Wang, G.X., Wang, Y., Amelin, Y., Tang, P., Liu, J.B., Jin, J., Huang, B., Wu, R.C. and Xue, S., 2019. An extremely brief end Ordovician mass extinction linked to abrupt onset of glaciation. *Solid Earth Sciences*, *4*(4), pp.190-198.

Myrow, P.M., Ramezani, J., Hanson, A.E., Bowring, S.A., Racki, G. and Rakociński, M., 2014. High-precision U–Pb age and duration of the latest Devonian (Famennian) Hangenberg event, and its implications. *Terra Nova*, *26*(3), pp.222-229.

Table S2. Superchron ages and references (ages, Ma). The superchrons are shown in chronological position with respect to the arm sequence. The latter is taken from the best fit arm location at 660 Myr galactic period with 165 Myr increments.

Superchron name	Superchron end (Ma)	Superchron start (Ma)	Arm sequence (Ma)	Superchron end (Ma)	Superchron start (Ma)	Superchron name
			52.05	83.1	119.7	Cretaceous long normal
			217.05	266.5	318.6	Kiaman
			382.05	458.4	477.7	Moyero
			547.05			
			712.05			
			877.05			
Maya, approximat e age	980	1020	1042.05	1084.0	1099.0	Portage (Keweenawan), may extend to 1070
			1207.05			

End Cretaceous long normal:

Wang, T., Ramezani, J. Wang, C., Wu, H., He, H. and Bowring, S.A. (2016). High-precision U-Pb geochronologic constraints on the Late Cretaceous terrestrial cyclostratigraphy and geomagnetic polarity from the Songliao basin, Northeast China. Earth and Planetary Science Letters, 446, 37-44.

Start Cretaceous long normal:

Leandro, C.G., Savian, J.F., Kochhann, M.V.L., Franco, D.R., Coccioni, R., Frontalini, F., Gardin, S., Jovane, L., Figueiredo, M., Tedeschi, L.R. and Janikian, L., 2022. Astronomical tuning of the Aptian stage and its implications for age recalibrations and paleoclimatic events. *Nature Communications*, *13*(1), p.2941.

End Kiaman:

Lanci, L., Galeotti, S., Ratcliffe, K., Tohver, E., Wilson, A. and Flint, S. (2022). Astronomically forced cycles in Middle Permian fluvial sediments from Karoo Basin (South Africa). Palaeogeography, Palaeoclimatology, Palaeoecology, 596, 110973.

Start Kiaman:

Hounslow, M.W. (2020). A geomagnetic polarity timescale for the Carboniferous. Lucas, Schneider et al. (eds) The Carboniferous Timescale, Spec. Publ. Geol. Soc. Lond.

Moyero:

Grappone, J.M., Chaffee, T., Isozaki, Y., Bauert, H. and Kirschvink, J.L. (2017). Investigating the duration and termination of the Early Paleozoic Moyero reversed polarity Superchron: Middle Ordovician paleomagnetism from Estonia, Palaeogeography, Palaeoclimatology, Palaeoecology (2017), doi: 10.1016/ j.palaeo.2017.07.024

Maya:

Pavlov, V.E., Gallet, Y. and Petrov, P.Y. (2019). A new Siberian record of the ~1.0 Gyr-old Maya superchron. Precambrian Research, 320, 350-370. https://doi.org/10.1016/j.precamres.2018.11.005

Portage Lake normal superchron. Keweenawan equivalent (Driscoll and Evans 2016):

Swanson-Hysell, N.L., Ramezani, J., Fairchild, L.M., Rose, I.R. (2019). Failed rifting and fast drifting: Midcontinent Rift development, Laurentia's rapid motion and the driver of Grenvillian orogenesis Geological Society of America Bulletin, 131(5-6). 10.1130/b31944.1
